# Enhancing the Mechanical Stability of 2D Fullerene with a Graphene Substrate and Encapsulation

**DOI:** 10.3390/nano13131936

**Published:** 2023-06-25

**Authors:** Taotao Yu, Jianyu Li, Mingjun Han, Yinghe Zhang, Haipeng Li, Qing Peng, Ho-Kin Tang

**Affiliations:** 1 School of Science, Harbin Institute of Technology (Shenzhen), Shenzhen 518055, China; 2 Shenzhen Key Laboratory of Advanced Functional Carbon Materials Research and Comprehensive Application, Harbin Institute of Technology (Shenzhen), Shenzhen 518055, China; 3 School of Materials Science and Physics, China University of Mining and Technology, Xuzhou 221116, China; 4 State Key Laboratory of Nonlinear Mechanics, Institute of Mechanics, Chinese Academy of Sciences, Beijing 100190, China; 5 School of Engineering Sciences, University of Chinese Academy of Sciences, Beijing 100049, China

**Keywords:** monolayer fullerene, fracture behavior, molecular dynamics simulation, tensile property, pre-crack system, graphene substrate

## Abstract

Recent advancements have led to the synthesis of novel monolayer 2D carbon structures, namely quasi-hexagonal-phase fullerene (qHPC_60_) and quasi-tetragonal-phase fullerene (qTPC_60_). Particularly, qHPC_60_ exhibits a promising medium band gap of approximately 1.6 eV, making it an attractive candidate for semiconductor devices. In this study, we conducted comprehensive molecular dynamics simulations to investigate the mechanical stability of 2D fullerene when placed on a graphene substrate and encapsulated within it. Graphene, renowned for its exceptional tensile strength, was chosen as the substrate and encapsulation material. We compared the mechanical behaviors of qHPC_60_ and qTPC_60_, examined the influence of cracks on their mechanical properties, and analyzed the internal stress experienced during and after fracture. Our findings reveal that the mechanical reliability of 2D fullerene can be significantly improved by encapsulating it with graphene, particularly strengthening the cracked regions. The estimated elastic modulus increased from 191.6 (qHPC_60_) and 134.7 GPa (qTPC_60_) to 531.4 and 504.1 GPa, respectively. Moreover, we observed that defects on the C60 layer had a negligible impact on the deterioration of the mechanical properties. This research provides valuable insights into enhancing the mechanical properties of 2D fullerene through graphene substrates or encapsulation, thereby holding promising implications for future applications.

## 1. Introduction

Carbon-based materials have garnered significant attention due to their versatile and promising applications, in particular, extensive research has focused on the exploration of two-dimensional (2D) carbon materials [1,2,3]. The excellent mechanical properties have been widely studied in many aspects, for example, the tensile strength on monolayer and bilayer graphene, the defect influence, the magic angle graphene and the phase transition induced by strain [4,5,6,7,8,9,10,11,12,13,14,15,16,17,18,19,20,21,22,23,24,25]. On the other hand, the fullerene system is one of the prominent candidates for future electronic applications as recently proposed in [26]. However, despite significant research efforts, a comprehensive understanding of the formation mechanism and stability of fullerene molecules remains elusive [27,28,29]. In a recent breakthrough, the experimental realization of a novel 2D carbon material exhibiting a semiconductor band gap of approximately 1.6 eV has been accomplished. This fabricated material can be fabricated into quasi-hexagonal-phase fullerene (qHPC_60_) and quasi-tetragonal-phase fullerene (qTPC_60_) structures [30]. The synthesis of these intriguing structures has opened new avenues for investigating their properties and exploring potential applications.

Many studies have been carried out on the mechanical properties and thermal stability of qHPC_60_ and qTPC_60_ [31,32,33,34,35,36]. Ying et al. comprehensively studied the properties of the newly synthesized monolayer qHPC_60_ film under axial tension using density functional theory (DFT) calculations and molecular dynamics (MD) simulations, using machine learning neuroevolutionary potentials [34]; the elasticity and fracture behavior of monolayer qHPC_60_ are found to be strongly anisotropic. Peng [37] carried out detailed DFT studies to compare the mechanical, kinetic, or thermodynamic stability of qTPC_60_ and qHPC_60_. Zhao et al. found through DFT that the ultimate tensile strength and fracture work of single-layer qHPC_60_ reached a maximum at 15 and 75°, respectively [38]. Shen et al. studied the thermodynamic stability of qHPC_60_, as well as its adhesive properties, ductility and mechanical properties [39]. Ribeiro et al. calculated the thermodynamic stability and fracture mode of qHPC_60_ and qTPC_60_ by MD [40]; the results showed that these structures have similar thermal stability, and the sublimation points are 3898 and 3965 K, respectively. qTPC_60_ undergoes a structural mutation after a critical strain threshold, breaking completely. The crack growth of qHPC_60_ (qTPC_60_) is linear (non-linear). The estimated elastic moduli of qHPC_60_ and qTPC_60_ are 175.9 and 100.7 GPa, respectively.

The effect of substrate and encapsulation on 2D fullerene is still not well explored, especially concerning its mechanical properties. The fullerene systems are also being found on the substrate or within the multi-layer heterostructure. An experiment reported mixing graphite oxide in toluene followed by heat treatment to obtain a multilayer structure consisting of graphene and fullerene layers [41]. Another study obtaining a film composed of a layer of closely packed fullerene sandwiched between two layers of graphene [42]; Young’s modulus of the material was more than an order of magnitude higher than that of the molecular fullerene. Mutual partial polymerization of fullerenes and partial polymerization of adjacent graphene flakes have also been studied [43]; the study showed that all considered compounds were energetically more stable when covalent bonds were formed between the components and that the cycloaddition reaction of fullerenes to fullerenes or graphene can be controlled using both pressure and temperature [44], or obtained under UV irradiation [45]. Graphene/fullerene/graphene sandwiches demonstrate switchable interfacial thermal resistance and show promising potential applications in switchable thermal devices [46].

**Figure 1:**
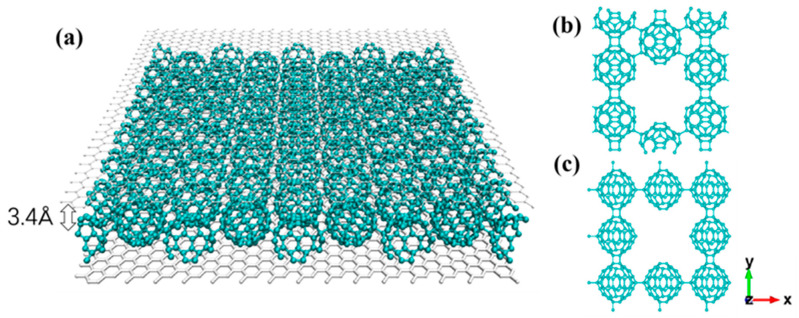
(**a**) Monolayer fullerene encapsulated with graphene sheets; (**b**) pre-cracked qHPC_60_; (**c**) pre-cracked qTPC_60_.

**Figure 2:**
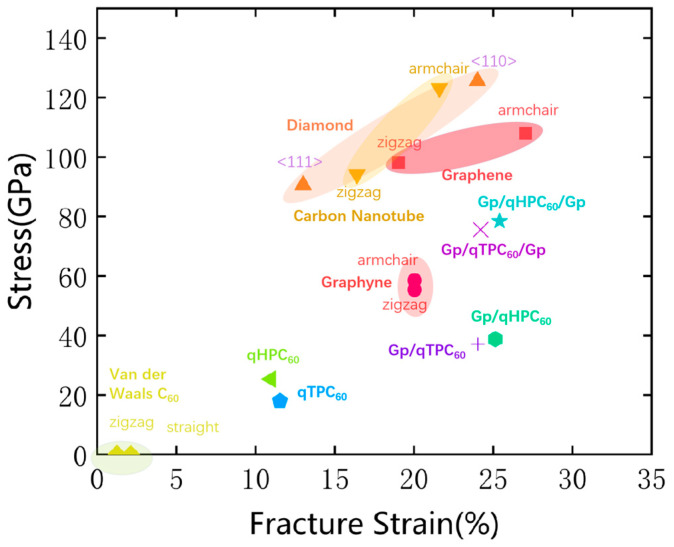
Comparison of the theoretical fracture strain and tensile strength of different carbon materials including van der Waals C_60_ [47], graphyne [48], single-wall carbon nanotubes [49], diamond [50], graphene [51,52]. Our proposed scheme of stabilizing 2D fullerene with graphene substrate and encapsulation are found to significantly enhance its tensile strength, leveraging the strength of the strong graphene layer.

We propose to enhance the tensile strength and mechanical stability of 2D fullerene, including qHPC_60_ and qTPC_60_, by using the substrate or encapsulation of graphene sheets, illustrated in Figure 1. As shown Figure 2, the tensile strength and mechanical stability of 2D fullerene has been greatly improved by using graphene sheet. Compared to other carbon materials, graphene-encapsulated 2D fullerene (Gp/qHPC_60_/Gp and Gp/qTPC_60_/Gp) has enhanced its tensile strength more than its monolayer counterpart, and the fullerene intermediate by the van der Waals force; however, it still has a smaller fracture tensile strength than a graphene sheet, carbon nanotube or diamond. The improved mechanical stability can potentially lead to a higher chance of employing qHPC_60_ or qTPC_60_ as a new generation of carbon functional nanomaterial.

In this article, we first outline our methods in Section 2. We test the mechanical stability of monolayer qHPC_60_ and qTPC_60_ of different system size, different strain rates, and different crack sizes in Section 3.1. We study how the use of a graphene substrate or encapsulation impact the mechanical stability of qHPC_60_ and qTPC_60_ in Section 3.2. We performed an internal stress analysis on qHPC_60_ with a substrate or encapsulated by graphene sheets in Section 3.3.

## 2. Materials and Methods

We carried out extensive MD simulations in LAMMPS (large-scale atomic/molecular massively parallel simulator) [53]. OVITO (open visualization tool) [54] and VMD (visual molecular dynamics) [55] were utilized to generate the atomistic simulation results and figures. We carried out ful atomistic MD simulations with the reactive force field ReaxFF (employing the parameter set for C/H/O [56,57]), a reactive potential allowing the formation and breaking of chemical bonds during fracture dynamics investigation. The simulation model of qHPC_60_ encapsulated with graphene sheets was established as shown in Figure 1. The distance between the graphene sheet and the fullerene layer is 3.4 Å [58]. All simulations were conducted at 300 K and zero pressure, using a simulation time step of 0.1 femtosecond (fs). Our calculation time was 500 fs. We used LAMMPS to calculate the stress of the materials, and its main theory is as follows. The stress tensor for atom *I* is given by the following formula, where *a* and *b* take on values *x*, *y*, *z* to generate the components of the tensor [59]:

$$S_{ab}=-mv_{a}-W_{ab}$$

The first term is a kinetic energy contribution for atom *I*. The second term is the virial contribution due to intra and intermolecular interactions, where the exact computation details are determined by the computation style. The virial contribution is:

$$\begin{array}{ll} W_{ab} & =\frac{1}{2}\sum_{n=1}^{N_{p}}\left(r_{1_{a}}F_{1_{b}}+r_{2_{a}}F_{2_{b}}\right)+\frac{1}{2}\sum_{n=1}^{N_{b}}\left(r_{1_{a}}F_{1_{b}}+r_{2_{a}}F_{2_{b}}\right)\\ & +\frac{1}{3}\sum_{n=1}^{N_{a}}\left(r_{1_{a}}F_{1_{b}}+r_{2_{a}}F_{2_{b}}+r_{3_{a}}F_{3_{b}}\right)+\frac{1}{4}\sum_{n=1}^{N_{d}}\left(r_{1_{a}}F_{1_{b}}+r_{2_{a}}F_{2_{b}}+r_{3_{a}}F_{3_{b}}+r_{4_{a}}F_{4_{b}}\right)\\ & +\frac{1}{4}\sum_{n=1}^{N_{i}}\left(r_{1_{a}}F_{1_{b}}+r_{2_{a}}F_{2_{b}}+r_{3_{a}}F_{3_{b}}+r_{4_{a}}F_{4_{b}}\right)+\mathrm{Kspace}\left(r_{i_{a}},F_{i_{b}}\right)+\sum_{n=1}^{N_{f}}r_{i_{a}}F_{i_{b}} \end{array}$$

The first term is a pairwise energy contribution where *n* loops over the *N_p_* neighbors of atom *I*, r_1_ and r_2_ are the positions of the 2 atoms in the pairwise interaction, and F_1_ and F_2_ are the forces on the 2 atoms resulting from the pairwise interaction. The second term is a bond contribution of similar form for the *N_b_* bonds which atom *I* is part of. There are similar terms for the *N_a_* angle, *N_d_* dihedral, and *N_i_* improper interactions atom *I* is part of. There is also a term for the KSpace contribution from long-range Coulombic interactions, if defined. Finally, there is a term for the *N_f_* fixes that apply internal constraint forces to atom *I*. The size of the equilibrated simulation box was 64 × 64 × 200 Å^3^. Our strain rate ranged from 10^8^ to 10^10^ s^−1^, and the final selected strain rate was 10^9^ s^−1^, with the tensile direction along the positive x-axis. We used the built-in calculation stress–strain command in LAMMPS to calculate the stress–strain in the x-direction. Our calculation time was 500 fs.

Young’s modulus, fracture stress and fracture strain were obtained from the simulated stress–strain curve. Young’s modulus was calculated as the initial slope of the stress–strain curve. Young’s modulus is the slope of the linear part of the stress–strain curve, taking the first 5% of the strain, while the fracture stress and fracture strain are defined at the point where the peak stress is reached. The total strain energy is defined as the area under the curve from the origin (0,0) to the breaking point. This is the energy that can be absorbed by the material before fracture, which is proportional to the area under the stress–strain curve.

## 3. Results and Discussion

### 3.1. Monolayer Fullerene

#### 3.1.1. Effect of System Size

**Figure 3:**
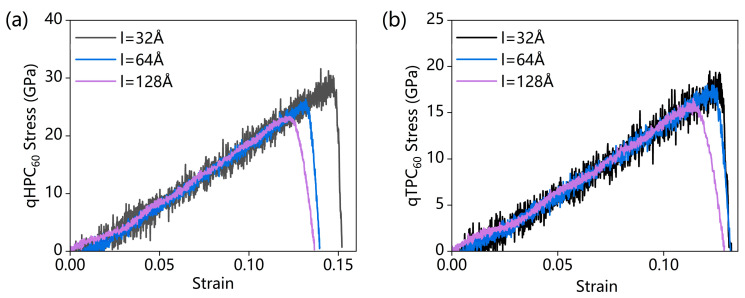
The tensile stress–strain curves for different system sizes; (**a**) qHPC_60_ and (**b**) qTPC_60_.

We obtained the stress–strain curves for monolayers of both qHPC_60_ and qTPC_60_ with different lattice sizes, from 32 Å × 32 Å to 128 Å × 128 Å, in which we employed a square box as the simulation cell in the x–y plane. As shown in Figure 3, the larger lattice size would lead to a smaller fracture stress in both qHPC_60_ and qTPC_60_. We found that this trend is consistent with the literature [34]. Based on the above analysis, our following experiments all selected 64 Å × 64 Å as the x–y plane size of the simulation cell to strike a balance between numerical precision and computational time.

#### 3.1.2. Effect of Strain Rate

**Figure 4:**
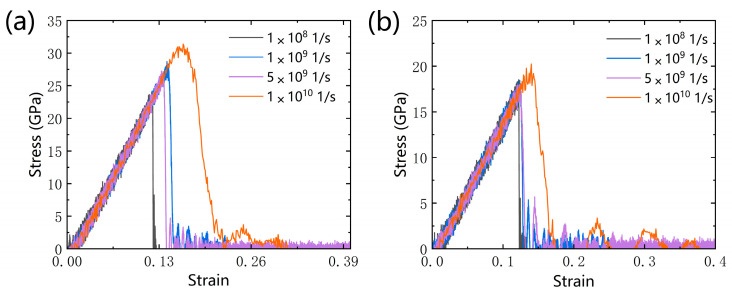
The tensile stress–strain curves for different strain rate; (**a**) qHPC_60_ and (**b**) qTPC_60_.

We investigated the tensile behavior of qHPC_60_ and qTPC_60_ at different strain rates. As shown in Figure 4a,b, the maximum stress and corresponding fracture strain increased with the increase in strain rate, indicating that single-layer qHPC_60_ and qTPC_60_ are more difficult to fracture at higher strain rates, and the bonds between the atoms are less prone to fracture. Through the above analysis, we chose a moderate strain rate of 1 × 10^9^ s^−1^ as the study fracture for other situations.

#### 3.1.3. Presence of Cracks

**Figure 5:**
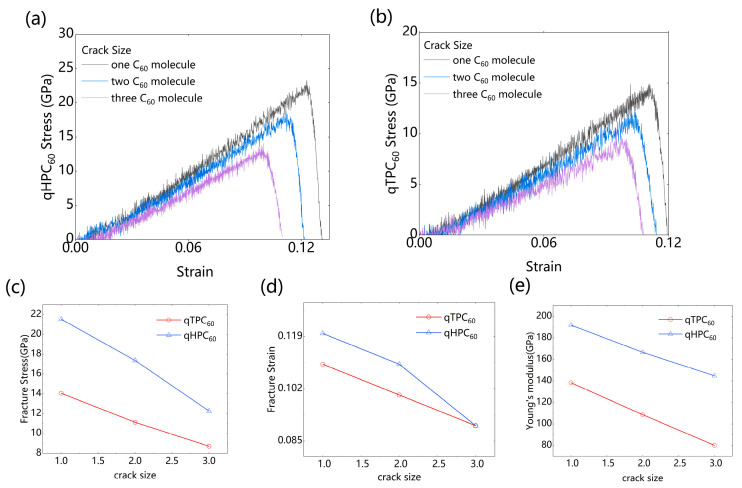
The effect of different cracks on the monolayer fullerene materials, including the stress–strain curves of (**a**) qHPC_60_ and (**b**) qTPC_60_, (**c**) fracture stress, (**d**) strain comparison, and (**e**) Young’s modulus ratio comparison.

We investigated the tensile behavior of qHPC_60_ and qTPC_60_ in the presence of a crack. The crack size was determined by the number of missing C_60_ molecules in the 2D fullerene layer. From Figure 5a,b, the maximum tensile stress continuously decreased with the increase in the crack size. The presence of pre-existing cracks in the 2D fullerene reduced its mechanical stability compared to the perfect lattice. As the crack size increased, the material’s elastic properties and fracture resilience deteriorated. In the linear elastic regime, the Young’s modulus of the monolayer qHPC_60_ and qTPC_60_ decreased from 191.8 (138.3 GPa) to 144.8 GPa (80.1 GPa), respectively, when increasing the crack size from one molecule to three molecules, as shown in Figure 5e. The crack size also influenced the fracture behavior, making the material more fragile. The fracture stress decreased from 21.5 (14.1 GPa) to 12.3 GPa (8.7 GPa), while the fracture strain decreased from 0.11 (0.12) to 0.09 (0.09).

### 3.2. qHPC_60_ and qTPC_60_ with Graphene Substrate

Through the analysis of the monolayer qHPC_60_ and qTPC_60_, we observed that the artificially synthesized monolayer 2D fullerene material was not very stable. Compared to the Young’s modulus of graphene, which is as high as 1000 GPa [59], the Young’s modulus of the 2D fullerene material was quite small. Therefore, to improve its tensile strength and stability we used graphene as a substrate for 2D fullerene.

**Figure 6:**
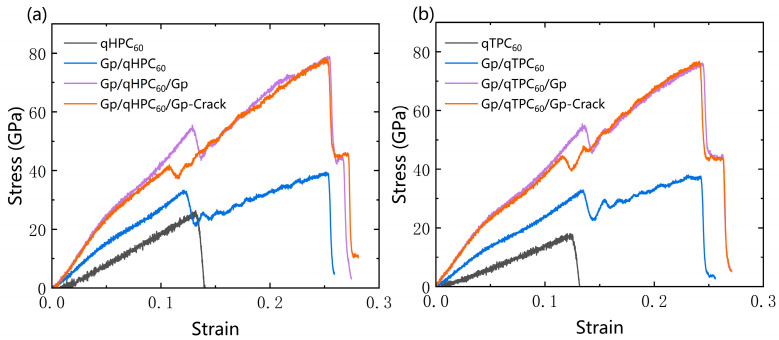
Comparison of the stress–strain curves of monolayer fullerene, (**a**) qHPC_60_ and (**b**) qTPC_60_, with a graphene substrate (Gp/qHPC_60_ and Gp/qTPC_60_), encapsulated with graphene sheets (Gp/qHPC_60_/Gp and Gp/qTPC_60_/Gp), and with cracks (Gp/qHPC_60_/Gp-Crack and Gp/qTPC_60_/Gp-Crack).

As can be seen from Figure 6a,b, both qHPC_60_ and qTPC_60_ of the substrate show improved stability. Moreover, the stability of the 2DC_60_ material becomes stronger when increasing the number of layers (these data can be seen in Table 1). From Table 2, we can see that the Young’s modulus, fracture stress and strain increases with the number of graphene layers, which is almost twice as much as the number of graphene substrate layers. This shows that adding a substrate to a single-layer fullerene material can increase its tensile mechanical stability. We also conducted substrate analysis on defective qHPC_60_ and qTPC_60_ and compared them with data from different substrates. We found that the time for the first fracture of defective fullerene was extended backwards, but the overall fracture stress, strain energy, and Young’s modulus decreased slightly. This suggests that the stability of the defective fullerenes, when “protected” by the graphene substrate, did not undergo significant changes as previously observed.

### 3.3. Analysis of Internal Atom Stress

#### 3.3.1. Without Defects

**Figure 7:**
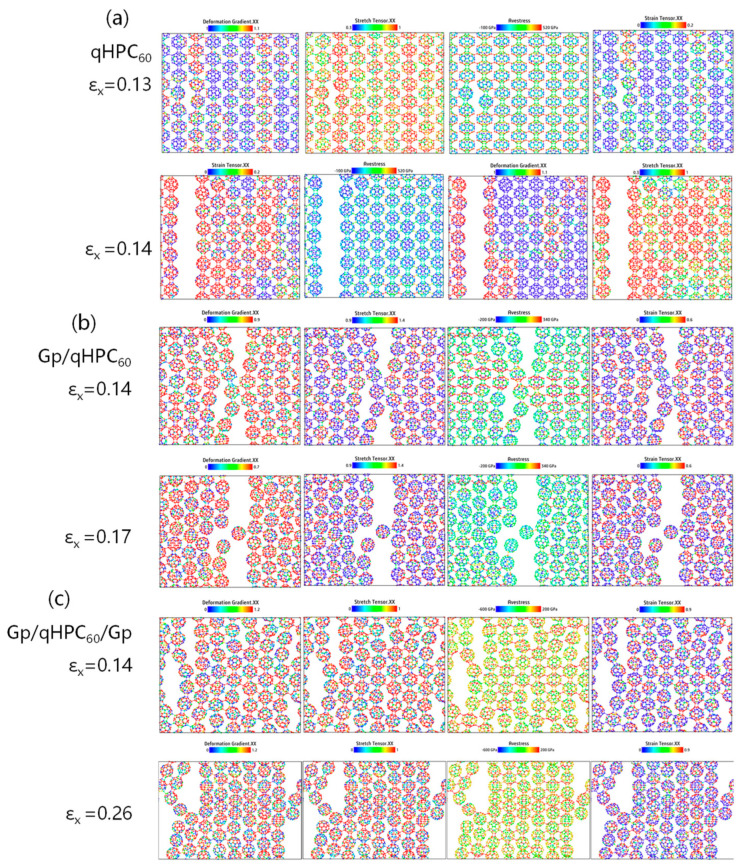
Fracture process of (**a**) the monolayer qHPC_60_, (**b**) with graphene substrate and (**c**) with graphene encapsulation.

To analyze the influence of substrates on the internal stress of atoms, we selected the more stable qHPC_60_ with a monolayer graphene substrate (Gp/qHPC_60_) and a bilayer graphene substrate (Gp/qHPC_60_/Gp) as the objects of analysis. We used the OVITO software to compare the tensile stress tensor, strain tensor, deformation gradient, and stress in the tensile direction (measured in Pa) before and after fracture, as shown in Figure 7. From Figure 7a, it can be observed that qHPC_60_ starts to fracture when the strain reaches 0.14. Stress concentrates in the tensile direction (the x-axis in this case), and the maximum positions of the tensile stress and strain tensors are mainly located at the fracture site, while deformation also concentrates around the fracture site.

After adding the substrate, the positions of stress concentration shift sequentially. Compared to qHPC_60_, both Gp/qHPC_60_ and Gp/qHPC_60_/Gp exhibit increased fracture strain, changing from the original 0.14 to 0.17 and 0.26, respectively. This indicates a longer time required for fracture, and the material’s resistance to deformation and tensile strength increases. Combined with the previous Young’s modulus and fracture stress data, this further supports the significant role of substrates in the tensile capability and stability of fullerene materials.

Therefore, we suggest appropriately increasing the substrate when using 2D fullerene materials in practical applications to enhance material stability. To analyze the effect of substrates on the internal stretching of atoms in 2D fullerene materials, we selected the more stable qHPC_60_ with a single-layer graphene substrate and a two-layer graphene substrate as the objects of analysis. We utilized the OVITO software to compare various parameters such as the tensile tensor, strain tensor, deformation gradient, and stress (in Pa) along the tensile direction during and after fracture, as illustrated in Figure 7.

From Figure 7a, it can be observed that the single-layer fullerene begins to fracture at a strain of 0.13 to 0.14. The strain tensor serves as a measure of local deformation, and the tensile stress is concentrated along the tensile direction (in this case, the x-axis). The tensile and strain tensors in the stretching direction mainly concentrate at the fracture position, and deformation is also localized at the fracture point. An increase in these quantities induces the formation of different types of defects, such as vacancies and cracks.

Upon the addition of a substrate, the stress concentration position shifts, and it is no longer like the single-layer qHPC_60_. The presence of a single- or double-layer graphene substrate protects the fullerene, resulting in an increased fracture strain from the original 0.14 to 0.17 and 0.26, respectively. This suggests that the fracture time becomes longer, and the material exhibits enhanced resistance to deformation and stretching, complementing the previously increased Young’s modulus. By comparing these key parameters during stretching and the changes that occur, it further demonstrates the significant role of the substrate in the tensile strength and stability of fullerene materials. Hence, in practical applications of 2D fullerene materials, the addition of a substrate is essential to enhance their tensile strength and stability.

#### 3.3.2. With Defects

From our previous analysis, we found that cracks have a greater impact on fullerene. When a fullerene molecule does not exist, the material is easy to fracture, and the Young’s modulus and fracture stress are continuously reduced. Therefore, even if only one fullerene molecule is missing, the tensile strength of fullerene will decrease.

**Figure 8:**
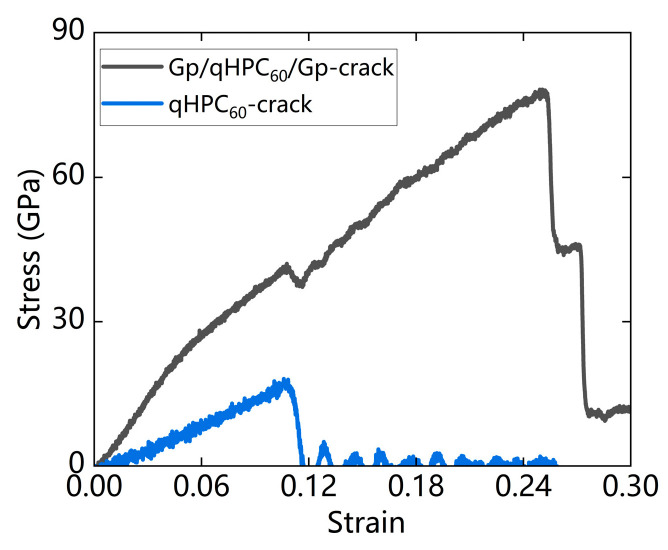
Comparison of stress–strain curves of single-layer qHPC_60_ with cracks (qHPC_60_-crack) and cracked qHPC_60_ with double-layer substrate (Gp/qHPC_60_/Gp-crack), (Black) with and (Blue) without being encapsulated between graphene sheets.

In this section, we compare the fullerene (GP/qHPC_60_/GP) on the strongest double-layer graphene substrate with the same single-layer qHPC_60_. Their stress–strain curves are shown in Figure 8. From the figure, it can be seen that the single-layer qHPC_60_ with a substrate fractured earlier. From Table 3, it can be seen that the monolayer qHPC_60_ with the substrate has a higher Young’s modulus and fracture stress, with the fracture stress increasing from 17.3 to 77.4 GPa, fracture strain increasing from 0.1 to 0.26, and Young’s modulus increasing from 107.1 to 518.7 GPa. This shows that the graphene substrate improves the tensile capacity of the single-layer qHPC_60_, and also makes the single-layer qHPC_60_ material more difficult to deform, making it more rigid and brittle.

**Figure 9:**
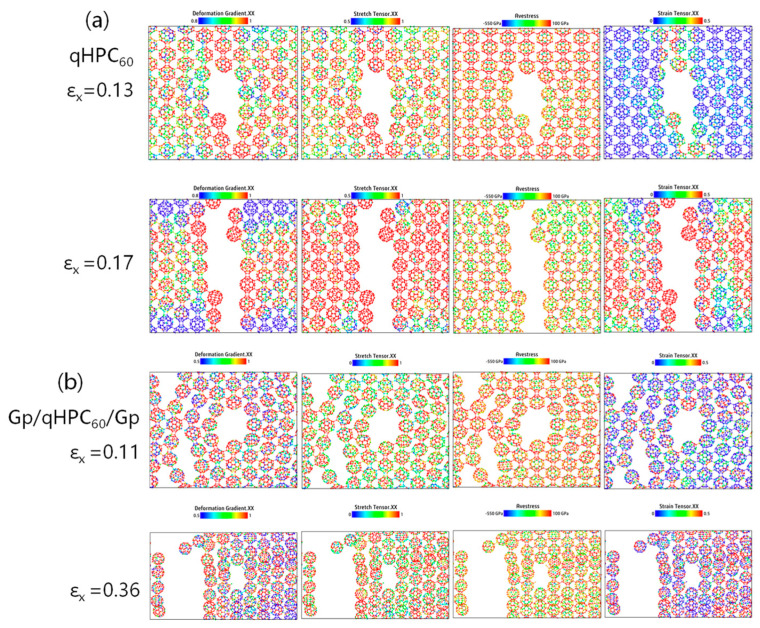
Fracture process of the pre-cracked qHPC_60_ (**a**) without and (**b**) with graphene encapsulation.

Further observations of the tensile nephogram results (Figure 9) show that when a monolayer of qHPC_60_ has cracks, the monolayer of qHPC_60_ (qHPC_60_-crack) without a substrate is not at the crack position but transferred to other positions compared with the monolayer of fullerene material with a bilayer of graphene substrate (GP/qHPC_60_/Gp-crack). This is because the graphene substrate slows the volume strain of the monolayer qHPC_60_. Combined with the tensile tensor, strain tensor and deformation gradient tensor in the figure, their values increase continuously during the tensile process, further indicating this point. The above analysis further shows that graphene is effective as the substrate of monolayer qHPC_60_, and can improve the mechanical stability and tensile capacity of monolayer qHPC_60_, attributed to the excellent mechanical properties of graphene.

## 4. Conclusions

In this study, we conducted a comprehensive investigation into the mechanical stability of 2D fullerene on a graphene substrate and under encapsulation using molecular dynamics simulations. We examined the presence of cracks on both qTPC60 and qHPC60, observing a slight degradation in the mechanical properties, including tensile strength, fracture stress and Young’s modulus, in the presence of cracks.

We compared the mechanical behaviors of qHPC_60_ and qTPC_60_ with and without the graphene substrate and encapsulation. Our results clearly demonstrate that encapsulating 2D fullerene with graphene significantly enhances its mechanical reliability, particularly in strengthening the cracked region. The estimated elastic modulus exhibited a substantial increase from 191.6 (qHPC_60_) and 134.7 GPa (qTPC_60_) to 531.4 and 504.1 GPa, respectively. Furthermore, we investigated the influence of cracks on the mechanical properties and examined the internal stress experienced during and after fracture. Interestingly, when encapsulated between graphene sheets, the position of the crack exhibited minimal imapct; on the other hand, in the absence of a substrate, the location of the initial fracture was highly determined by the crack position.

For future research directions, it would be advantageous to enhance the precision and scale of our molecular dynamics study by incorporating machine learning neuroevolutionary potentials [34]. Moreover, as both the inter-fullerene carbon single bonds and [2 + 2] cycloaddition bond exist, the orientation of the 2D fullerene would have great impact on the mechanical properties [34,37,38], which is worth further investigation in the system with graphene substrate. Furthermore, considering that C_60_ often exists in a multilayer form, investigating the mechanical stability of multilayer C_60_ warrants further exploration.

## Figures and Tables

**Table 1:** Comparison of the fracture stress, strain energy and Young’s modulus for qHPC_60_ and its associated structures.

Substrate	Fracture Stress (GPa)	Strain Energy (J/m^3^)	Young’s Modulus (GPa)
qHPC_60_	24.5	1.6	191.6
Gp/qHPC_60_	39.5	6.1	322.7
Gp/qHP C_60_/Gp	78.7	11.4	531.4
Gp/qHPC_60_/Gp-Crack	77.4	11.1	518.7

**Table 2:** Comparison of the fracture stress, strain energy and Young’s modulus for qTPC_60_ and its associated structures.

Substrate	Fracture Stress (GPa)	Strain Energy (J/m^3^)	Young’s Modulus (GPa)
qTPC_60_	17.6	1.1	134.7
Gp/qTPC_60_	37.4	5.7	295.9
Gp/qTPC_60_/Gp	76.7	10.7	504.1
Gp/qTPC_60_/Gp-Crack	75.3	10.2	489.1

**Table 3:** Comparison of fracture stress and Young’s modulus data of single-layer qHPC_60_ with cracks (qHPC_60_-crack) and cracked qHPC_60_ with double-layer substrate (Gp/qHPC_60_/Gp-crack).

Substrate	Fracture Stress (GPa)	Fracture Strain	Young’s Modulus (GPa)
qHPC_60_-crack	17.3	0.12	170.1
Gp/qHPC_60_/Gp-crack	77.4	0.36	518.7

## Data Availability

The data presented in this study are available on request.
